# Pharmacognostic and antifungal investigations of *Elaeocarpus ganitrus (Rudrakasha)*

**DOI:** 10.4103/0250-474X.65021

**Published:** 2010

**Authors:** B. Singh, A. Chopra, M.P.S. Ishar, A. Sharma, T. Raj

**Affiliations:** Pharmacognosy and Phytochemistry Laboratory, Department of Pharmaceutical Sciences, Guru Nanak Dev University, Amritsar-143 005, India; 1 University Institute of Pharmaceutical Sciences, Panjab University, Chandigarh-160 014, India

**Keywords:** *Rudrakasha*, *Elaeocarpus ganitrus*, antifungal activity, *Candida albicans*, *Aspergillus niger*

## Abstract

*Rudrakasha* is the dried bead obtained from the ripe fruit of *Elaeocarpus ganitrus* Roxb. (Family: Elaeocarpaceae). Microscopic studies revealed the presence of a hard endocarp with lignified isodiametric sclereids, seeds with membranous seed coat, which enclosed a dense cellular endosperm comprising of calcium oxalate druses. Physicochemical parameters showed that total ash was 1.36 times and 1.56 times more than the acid insoluble ash and water-soluble ash, respectively. Further, ethanol had a maximum extractable value of 2.4% and moisture content was found to be 9.7%. Different extracts, petroleum ether, chloroform, ethanol and water were prepared. Chemically the extracts showed the presence of phytosterols, fats, alkaloids, flavonoids, carbohydrates, proteins and tannins. The extracts were evaluated for antifungal activity on different fungal strains. Chlorofom and ethanol extracts have high antifungal activity against *Candida albicans*. Whereas, chloroform, ethanol and water extracts showed moderate inhibition against *Aspergillus niger*.

*Elaeocarpus ganitrus* Roxb. (Syn. *E. sphaericus* Gaertn; family Elaeocarpaceae), is popular for its attractive fruit stones. Its beads are covered by an outer shell of blue color on fully ripening, hence also called blueberry beads[1]. It finds a prominent place in Hindu religion and Ayurveda, the ancient Indian system of medicine. In Hindi it is known as *Rudraksha*[2]. *Rudrakasha* fruits are thermogenic, sedative and are useful in cough, bronchitis, neuralgia, cephalagia, anorexia, migraine, manic conditions and other brain disorders[3]. The flesh or pulp of drupe is given in epilepsy, diseases of head and in mental illness[4]. Besides it is reported to exhibit multifarious pharmacological activities that include antiinflammatory[5], analgesic[6], sedative[6], antidepressant[7], antiasthmatic[8], hypoglycemic[4], antihypertensive[9–11], smooth muscle relaxant[12], hydrocholeretic[12], antiulcerogenic[7] and anticonvulsant[13]. A detailed study on pharmacognostic standards of *Rudraksha* is lacking. In ancient Indian medicine, the fruits were employed to ward off evil spirits and omens[14] which can be considered to be at least partly manifestations of microbial infection. In addition, the *Rudrakasha* extracts also known to exhibit very high antimicrobial activity[15]. Therefore, it was decided to evaluate the drug (beads) for various pharmacognostic parameters and beads extracts for antifungal activity against different fungal strains.

Dried fruits (beads) of *Elaeocarpus ganitrus* were procured from a cultivated source, Hind Herb Store, Saharanpur, Uttar Pradesh, India, in the month of September 2007. Identity of dried fruit was confirmed through Plant Anatomy Research Centre, Medicinal Plant Research Unit, Chennai. Voucher specimen no. PARC/2008/164 has been deposited in the herbarium of the same institute. Coarsely powdered dried fruits were successively extracted with petroleum ether, chloroform and ethanol for 48 h each using Soxhlet apparatus and finally boiled with distilled water for 6 h. The extracts were filtered, concentrated *in vacuo* and dried in an oven at 40-50°. After removal of solvents under *vacuo* from various extracts, the percentage of various extractives obtained was: petroleum ether extract (PE) 1.655, chloroform extract (CE) 0.458, ethanol extract (EE) l.538 and water extract (WE) 1.013. Other physicochemical parameters[16,17] showed total ash of 1.5%, acid insoluble ash 1.1%, water-soluble ash 0.96%, pet ether soluble extractive 1.3%, chloroform soluble extractive 0.5%, ethanol soluble extractive 2.4%, water soluble extractive 1.8% and loss on drying 9.7%. Phytochemical screening[18–22] gave positive test for phytosterols, fats, alkaloids, flavonoids, carbohydrates, proteins and tannins.

For microscopic studies the paraffin embedded specimens were sectioned with the help of rotary microtome. Thickness of the sections was 10-12 μm. De-waxing of the sections was done by customary procedure[23]. The sections were stained with toluidine blue as per well documented method[24]. Wherever necessary sections were also stained with safranin, fast-green and iodine solution. Powdered material was cleared with chloral hydrate (S. D. Fine Chemicals Ltd., Mumbai, India) and mounted in glycerine (Glaxosmithkline Pharmaceuticals Ltd. Mumbai, India) and stained where ever necessary. The fruit had a hard, stony endocarp or sclerotesta. The surface of the endocarp was black, deeply folded, and hard with ridges and furrows. Mesocarp was not seen in the fruit. The fruit included five or six carpels, each carpel having a single large seed (fig. 1). In sectional view, the seed was elliptical and consisted of a membranous seed coat. The seed coat enclosed a dense cellular endosperm (fig. 2). The seed coat, though membranous, is differentiated into outer darkly staining cell layer and inner vertically oblong compact dark cell layer. They have dense accumulation of tannins. The middle seed coat has two or three layers of circular brachysclereids or stone cells. Their walls are highly lignified. The sclereids have dark cell contents. The endosperm cells are in parallel compact rows. They extend from periphery to the centre; the cells are squarish and thin walled. The cells towards the periphery are smaller and they become gradually larger towards the centre of the seed. The endosperm cells have large calcium oxalate druses or sphaerocrystals. The crystals are either one or two per cell. They are random in distribution (fig. 3). The druses are 10 μm in diameter. The stony endocarp or sclerotesta consists of only sclereids. No other cell types or cell inclusions are evident in the powder (fig. 4). The sclereids may be short or elongated. The short sclereids are isodiametric. The sclereid walls are highly lignified; the cells have wide lumen with brownish content.

**Fig. 1:**
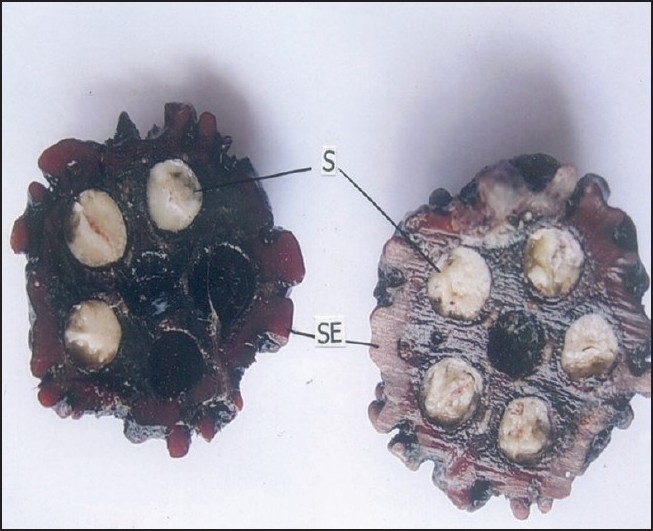
TS of stony endocarp of *E. ganitrus* fruit Transverse section of the stony endocarp of *E. ganitrus* fruit showing S seeds and SE stony endocarp

**Fig. 2:**
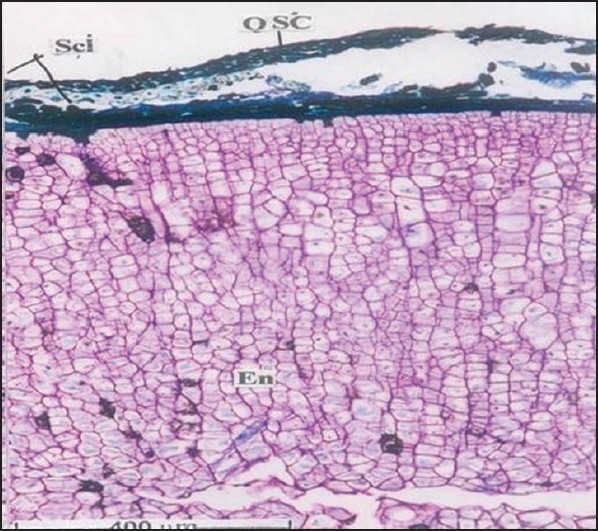
TS of *E. ganitrus* seed Transverse section of E. ganitrus seed showing En- endosperm, Fufunicle, Isc- inner seed coat and Osc- outer seed coat

**Fig. 3:**
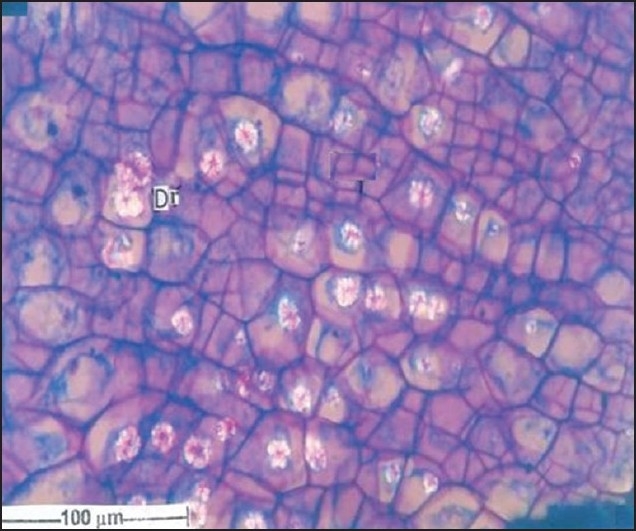
TS of *E. ganitrus* seed showing calcium oxalate druses Transverse section of *E. ganitrus* seed with calcium oxalate druses (Dr) in the endosperm

**Fig. 4:**
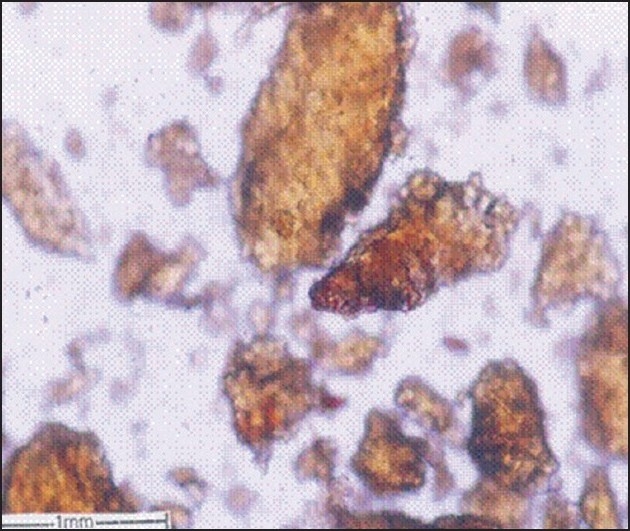
TS of *E. ganitrus* seed showing thick masses of sclereids

*In vitro* antifungal activity of all the extracts was carried out using the disk-diffusion assay[25] and broth dilution test[26,27]. The disk-diffusion assay was applied to determine the growth inhibition of fungi by extracts to be tested. Overnight fungal cultures (100 µl) were spread onto SDA. The extracts were applied to 8 mm disks (Whatman paper No.1). After 48 h of incubation at 25°, the diameter of growth inhibition zones was measured. MIC of all extractives was determined by broth dilution test which was performed in test tubes. The conidial suspension, which gave the final concentration of 1×10^5^ CFU/ml, was prepared. A growth control tube and a sterility control tube were used in each test. After 24-72 h incubation at 25°, the MIC was determined visually as the lowest concentration that inhibits growth, evidenced by the absence of turbidity on the fungal strains, *Asperagillus niger* (MTCC-281), *Candidum geotrichum* (MTCC-3993), *Candida albicans* (MTCC-227), *C. glabrata* (MTCC-1637) and *C. tropicalis* (MTCC-230) using ketoconazole as the positive control. Minimum inhibitory concentration (MIC) is the concentration required to inhibit fungal cell proliferation by 50% after exposure of cells to test compounds. Inhibitory concentration in terms of MIC (mg/ml) was determined using turbidimetry method (Table 1). Maximum inhibition was observed for CE (MIC 1.5 mg/ml), followed by EE (MIC 4.0 mg/ml) on *C. albicans*. In the case of *C. tropicallis* maximum inhibition of MIC 5.0 mg/ml was observed for CE, whereas, no inhibition was observed for EE and WE. Maximum inhibition of MIC 3.0 mg/ml on *A. niger* was observed for CE and EE, which is followed by WE (MIC 5.0 mg/ml). It is also pertinent to mention here that various plant extracts showed no sign of inhibition on *C. glabrata* and *G. candidum* even at higher concentration.

**TABLE 1:** *IN VITRO* ACTIVITY OF *E. GANITRUS* ON VARIOUS FUNGAL STRAINS

Strains	Extractives	Dilutions (mg/ml)								
		0.125	0.5	1.0	1.5	2.0	3.0	4.0	5.0	Ketocon-azole
*C. albicans*	CE	+++	++	+	‐	‐	‐	‐	‐	‐
MTCC 3017	EE	+++	+++	+++	+++	++	+	‐	‐	‐
	WE	+++	+++	+++	+++	+++	+++	+++	+++	‐
*C. tropicallis*	CE	+++	+++	+++	+++	+++	++	+	‐	‐
MTCC 230	EE	+++	+++	+++	+++	+++	+++	+++	+++	‐
	WE	+++	+++	+++	+++	+++	+++	+++	+++	‐
*C. glabrata*	CE	+++	+++	+++	+++	+++	+++	+++	+++	‐
MTCC 1637	EE	+++	+++	+++	+++	+++	+++	+++	+++	‐
	WE	+++	+++	+++	+++	+++	+++	+++	+++	‐
*C. geotricum*	CE	+++	+++	+++	+++	+++	+++	+++	+++	‐
MTCC 3993	EE	+++	+++	+++	+++	+++	+++	+++	+++	‐
	WE	+++	+++	+++	+++	+++	+++	+++	+++	‐
*A. niger*	CE	+++	+++	++	++	+	‐	‐	‐	‐
MTCC 1344	EE	+++	+++	++	++	+	‐	‐	‐	‐
	WE	+++	+++	+++	++	++	+	+	‐	‐

*+++ Highly turbid, ++ moderately turbid, + weakly turbid and ‐ No turbidity. The microorganisms tested were, *Candida albicans, C. tropicalis, Candida glabrata, Candidum geotrichum* and *Asperagillus niger*

In conclusion, microscopic studies revealed the presence of a hard stony endocarp and elliptical seed with a membranous seed coat, which enclosed a dense cellular endosperm and had a dense layer of circular brachysclereids. Endosperm had a large calcium oxalate druses or spaheorocrystal. Powder microscopy revealed that stony endocarp had only isodiametric sclereids, with lignified walls. Physicochemical parameters viz total ash, acid insoluble ash; water soluble ash, extractive values and loss on drying were performed. Total ash was about 1.36 times more than the acid insoluble ash indicating the presence of good acid soluble inorganic matter in *E. ganitrus*. The water soluble ash was 1.56 times less than the total ash. Out of all the solvents used, ethanol had a maximum extractable value of 2.4%, whereas chloroform had a minimum value of 0.5%. Moisture content was found to be 9.7%. In phytochemical investigations, PE showed the presence of phytosterols along with fats and fixed oils. CE had phytosterols. EE gave the tests for alkaloids, flavonoids, carbohydrates, proteins and tannins. WE showed the presence of proteins, tannins and carbohydrates. All the extracts (PE, CE, EE and WE) were evaluated for the antifungal activity on different fungal strains. The maximum inhibition (MIC 1.5 mg/ml) was observed for CE against *C. albicans*. The CE and EE showed maximum inhibitory potential (MIC 3.0 mg/ml) on *A. niger*. In conclusion the CE and EE were found to be more active antifungals. The activity guided fractionation of active extracts may be taken up for further development.
